# Supplementary material for: Patterns of Intron Gain and Loss in Fungi
Source: PLoS Biol. 2004 Nov 30;2(12):e422. doi: 10.1371/journal.pbio.0020422 (PMC532390; doi:10.1371/journal.pbio.0020422)
Supplement: Table S1 — Also available at http://genes.mit.edu/NielsenEtAl/. (4.3 MB ZIP). [file pbio.0020422.st001.zip › NielsenEtAl/html/298.html]

AN8868.1.NCU01444.1.MG06914.1.FG07473.1


```
 CLUSTAL W (1.82) Multiple Sequence Alignments - Introns Inserted


Sequence 1: NCU01444.1	188 aa
Sequence 2: FG07473.1	186 aa
Sequence 3: MG06914.1	188 aa
Sequence 4: AN8868.1	187 aa
Alignment Length: 190 aa
Number Identitical Residues: 114 aa
Alignment Score (without introns) 5058


MG06914.1 	MAIT--KQRKIAIVGSRSVG1KSSLAVRFVDGHFVESYYPTIENTFSKEITYKNQIYSTE
NCU01444.1	MPSPATKQRKIAIVGSRSVG1KSSLAVRFVDGHFVESYYPTIENTFSKEIRWKNQDYSTE
FG07473.1 	MPSP--KQRKVAIVGSRSVG1KSSLAVRFVDGHFVDSYYPTIENTFSKMIKYKGQDYSTE
AN8868.1  	MPSAP-KQRKIAIVGSRSVG1KSSLTVRFVEHHFVESYYPTIENTFSQIIKYNGQDYATE
          	*. .. ****:********* ****:****: ***:***********: * ::.* *:**

MG06914.1 	IVDTAGQ~DEYSILNSKHFVGIHGYIIVYSVGSLTSFEMVRTIRDKIIHHL0GVAEGDVP
NCU01444.1	IVDTAGQ0DEYSILNSKHFIGIHGYMLVYSVSSLASFEMVQVIRDKILNHL0GTDN--VP
FG07473.1 	IVDTAGQ~DEYSILNSKHFIGIHGYMLVYSVSSLPSFEMVQVIREKILNHL0GTDS--VP
AN8868.1  	IVDTAGQ0DEYSILNSKHFIGIHGYIIAYSVASRQSFDMVRVIRDKILNHL0GADY--VP
          	******* ***********:*****::.***.*  **:**:.**:**::** *.    **

MG06914.1 	IVIVGNKSDLRPEQRQVTPQDGQALATELKCGYTEASARYDENVAQAFELMIARIEGAPN
NCU01444.1	ICIVGNKSDLRPEQRQVTPEDGKALAEKYKCAWTEASARYNENVARAFELLIGEVEKSNN
FG07473.1 	ICIVGNKSDLRPEQRQVTPEDGQKLSEKIQCGWTEASARYNENVGKAFELLIGQIEKSQN
AN8868.1  	LVVVGNKSDLKPEQRQVSLDEGRQLGEEFRCAFTEASARLDFNVARAFELMIGEIEKSQN
          	: :*******:******: ::*: *. : :*.:****** : **.:****:*..:* : *

MG06914.1 	GGEATNGGKCAVM
NCU01444.1	PTATNENGKCVVM
FG07473.1 	PGEAPAKSNCILM
AN8868.1  	PSQPTGGNKCALM
          	   .   .:* :*
```
